# Endoscopic papillary large balloon dilation with a novel non-slip balloon in a patient with surgically altered anatomy

**DOI:** 10.1055/a-2499-7613

**Published:** 2025-01-14

**Authors:** Haruo Miwa, Yuichi Suzuki, Kazuki Endo, Ritsuko Oishi, Hiromi Tsuchiya, Kazushi Numata, Shin Maeda

**Affiliations:** 1 26437 Gastroenterological Center, Yokohama City University Medical Center, Yokohama, Japan; 2 Department of Gastroenterology, Yokohama City University Graduate School of Medicine, Yokohama, Japan

Endoscopic papillary large balloon dilation (EPLBD) in balloon enteroscopy-assisted endoscopic retrograde cholangiopancreatography (BE-ERCP) is an effective procedure for stone removal 1 2 . However, balloon slipping often occurs when an enteroscope is unstable. A novel balloon catheter (RIGEL Balloon Dilation Catheter; Japan Lifeline Co., Ltd., Tokyo, Japan) forms a waist because of the central band, which can prevent slipping during dilation ( Fig. 1 ) 3 4 . Herein, we report a case of a patient with surgically altered anatomy who underwent EPLBD with a 12-mm RIGEL balloon ( Video 1 ).

**Fig. 1:**
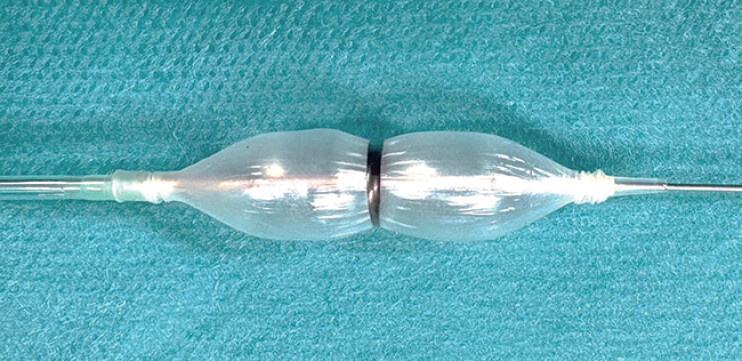
A novel balloon forms a waist because of the central band, which can prevent slipping during dilation.

The RIGEL balloon was effective for endoscopic papillary large balloon dilation without slippage, even when enteroscope positioning was unstable in a patient with surgically altered anatomy.Video 1

A 60-year-old male who had undergone distal gastrectomy with Roux-en-Y reconstruction was referred to our hospital for choledocholithiasis. BE-ERCP was performed to remove common bile duct stones ( Fig. 2 ). A short-type single-balloon enteroscope (SIF-H290S; Olympus Medical Systems, Tokyo, Japan) was inserted, and the papilla of Vater was detected in the third part of the duodenum. A rotatable papillotome was used for biliary cannulation; however, alignment for biliary access was difficult. The double-guidewire technique was used because the enteroscope was unstable. Eventually, selective biliary cannulation was achieved. Cholangiography revealed gallstones and a dilated distal bile duct. The EPLBD was attempted to facilitate recanalization and stone removal ( Fig. 3 ). A RIGEL 12-mm balloon was inserted along the guidewire smoothly, and a black marker at the middle part of the balloon was adjusted at the orifice of the bile duct. Subsequently, the balloon was gradually inflated, and delayed dilation of the middle part prevented balloon slippage. The balloon was dilated up to the diameter of the bile duct. Finally, a biliary stent was placed because the procedure took a long time. The patient was discharged without complications and stone removal was scheduled for a later date.

**Fig. 2:**
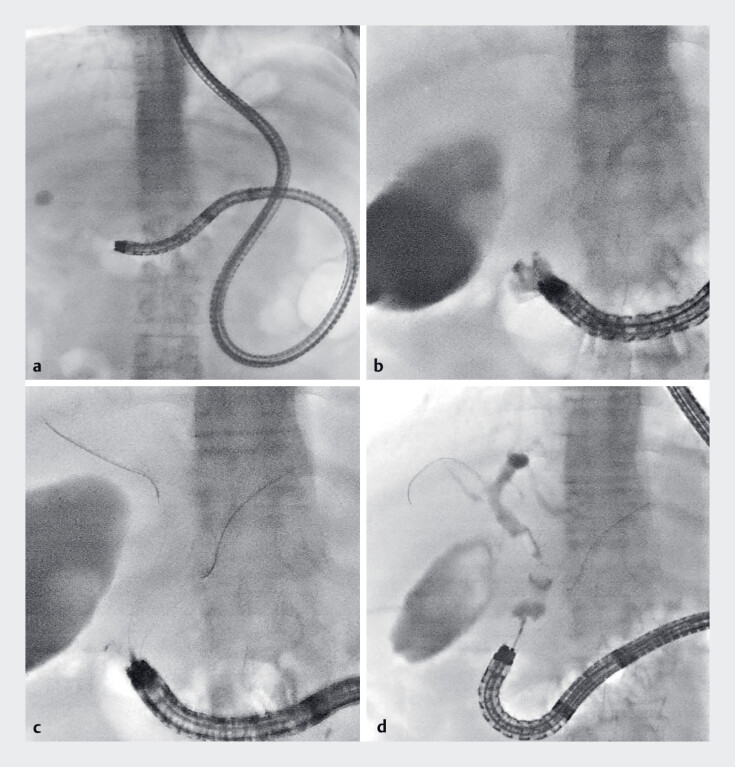
Balloon enteroscopy-assisted endoscopic retrograde cholangiopancreatography. **a** A short-type single balloon enteroscope reached the papilla of Vater. **b** Selective biliary cannulation was difficult. **c** The double-guidewire technique facilitated guidewire insertion into the bile duct. **d** Cholangiography showed dilated bile duct and gallstones.

**Fig. 3:**
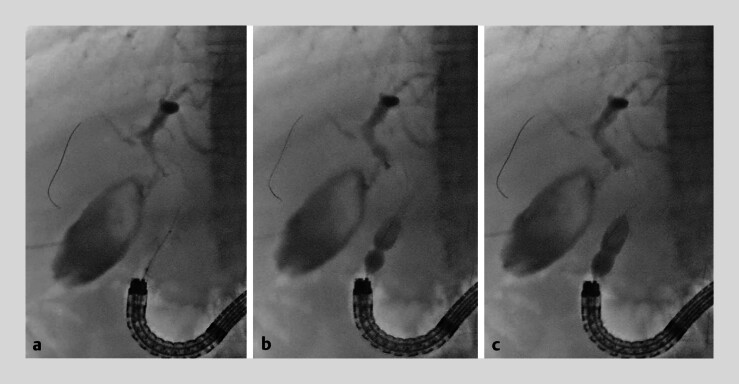
Endoscopic papillary large balloon dilation (EPLBD). **a** The balloon catheter is inserted across the papilla. **b** The waist of the RIGEL prevents balloon slippage during dilation. **c** EPLBD is successfully performed up to the diameter of the bile duct.

To the best of our knowledge, this is the first report of an EPLBD using a novel 12-mm RIGEL balloon. The unique structure of the RIGEL balloon was effective for EPLBD without slippage, even when enteroscope positioning was unstable.

Endoscopy_UCTN_Code_TTT_1AR_2AH

## Notes

Endoscopy E-Videos https://eref.thieme.de/e-videos
*E-Videos* is an open access online section of the journal *Endoscopy* , reporting on interesting cases and new techniques in gastroenterological endoscopy. All papers include a high-quality video and are published with a Creative Commons CC-BY license. Endoscopy E-Videos qualify for HINARI discounts and waivers and eligibility is automatically checked during the submission process. We grant 100% waivers to articles whose corresponding authors are based in Group A countries and 50% waivers to those who are based in Group B countries as classified by Research4Life (see: https://www.research4life.org/access/eligibility/ ). This section has its own submission website at https://mc.manuscriptcentral.com/e-videos .
